# Cell-Free Synthetic Glycobiology: Designing and Engineering Glycomolecules Outside of Living Cells

**DOI:** 10.3389/fchem.2020.00645

**Published:** 2020-07-29

**Authors:** Thapakorn Jaroentomeechai, May N. Taw, Mingji Li, Alicia Aquino, Ninad Agashe, Sean Chung, Michael C. Jewett, Matthew P. DeLisa

**Affiliations:** ^1^Robert Frederick Smith School of Chemical and Biomolecular Engineering, Cornell University, Ithaca, NY, United States; ^2^Graduate Field of Biochemistry, Molecular and Cell Biology, Cornell University, Ithaca, NY, United States; ^3^Department of Chemical and Biological Engineering, Northwestern University, Evanston, IL, United States; ^4^Center for Synthetic Biology, Northwestern University, Evanston, IL, United States; ^5^Chemistry of Life Processes Institute, Northwestern University, Evanston, IL, United States

**Keywords:** Cell-free system, chemoenzymatic synthesis, glycoscience, glycoprotein therapeutics and vaccines, metabolic glycoengineering, post-translational modification, nucleotide sugars, synthetic biology

## Abstract

Glycans and glycosylated biomolecules are directly involved in almost every biological process as well as the etiology of most major diseases. Hence, glycoscience knowledge is essential to efforts aimed at addressing fundamental challenges in understanding and improving human health, protecting the environment and enhancing energy security, and developing renewable and sustainable resources that can serve as the source of next-generation materials. While much progress has been made, there remains an urgent need for new tools that can overexpress structurally uniform glycans and glycoconjugates in the quantities needed for characterization and that can be used to mechanistically dissect the enzymatic reactions and multi-enzyme assembly lines that promote their construction. To address this technology gap, cell-free synthetic glycobiology has emerged as a simplified and highly modular framework to investigate, prototype, and engineer pathways for glycan biosynthesis and biomolecule glycosylation outside the confines of living cells. From nucleotide sugars to complex glycoproteins, we summarize here recent efforts that harness the power of cell-free approaches to design, build, test, and utilize glyco-enzyme reaction networks that produce desired glycomolecules in a predictable and controllable manner. We also highlight novel cell-free methods for shedding light on poorly understood aspects of diverse glycosylation processes and engineering these processes toward desired outcomes. Taken together, cell-free synthetic glycobiology represents a promising set of tools and techniques for accelerating basic glycoscience research (e.g., deciphering the “glycan code”) and its application (e.g., biomanufacturing high-value glycomolecules on demand).

## Introduction

Glycans and glycan-modified biomolecules including glycolipids, glycoproteins, and glycosylated small molecules (collectively referred to as glycomolecules hereafter) are ubiquitous across all domains of life. It has now been firmly established that conjugation of carbohydrate structures to biomolecules including lipids, proteins, metabolites, and nucleic acids profoundly affects their physicochemical properties, subcellular localization, immunogenicity, and pharmacokinetics and pharmacodynamics (Walt, 2012; Varki, 2017a,b). Moreover, the redesign of carbohydrate structures on glycomolecules has been demonstrated to improve their therapeutic properties such as extending half-life *in vivo* (Elliott et al., 2003; Chen et al., 2012), fine-tuning efficacy (Jefferis, 2009a), and enhancing vaccine-specific immunity (Berti and Adamo, 2018; Stevenson et al., 2018). At present, however, challenges associated with preparing structurally-homogeneous glycomolecules at sufficient quantities has limited our fundamental understanding of glycosylation processes and their corresponding biotechnological applications. Naturally occurring glycans are usually complex, exist in small quantities, and are present as heterogeneous mixtures or glycoforms. This heterogeneity is due to the fact that glycan biosynthesis is not template driven like those of nucleic acid and protein synthesis, but rather through a series of glycosylation reactions catalyzed by specific glycosyltransferase (GT) enzymes that are co-expressed in different subcellular locations (Aebi, 2013). Such processes are highly dynamic, resulting in multiple glycan structures on the glycomolecules (Varki and Kornfeld, 2015). Further complexity is added to the glycan repertoire through branching of the glycan core, the addition of terminal sugars such as sialic acids, as well as the modification of carbohydrates with functional groups such as phosphate, sulfate, and acetate. In addition, as glycosylation is essential for viability and highly regulated within eukaryotic cells, small perturbations in the glycosylation network can severely reduce cell fitness, further complicating glycoengineering approaches in certain living organisms (Clausen et al., 2015).

## Synthetic Glycobiology

The term “synthetic glycobiology” was first used to describe the redesign of GT assembly lines for the production of specific glycan structures using protein engineering and chemical approaches (Czlapinski and Bertozzi, 2006). This initial definition referred narrowly to the exploitation of Golgi-resident GTs to engineer protein glycosylation inside and on the surface of eukaryotic cells, as exemplified by a number of notable glycoengineering studies in yeast (Choi et al., 2003; Hamilton et al., 2003) and more recently in mammalian cells (Meuris et al., 2014; Chang et al., 2019). These successes notwithstanding, simpler, cell-viability independent systems that permit bottom-up assembly of prescribed glycosylation pathways and controllable biosynthesis of designer glycomolecules are of great scientific and technological interest, and have the potential to be transformative. In this vein, Aebi and coworkers pioneered the first bacterial glycoprotein expression platform by transferring the *N-*linked glycosylation machinery from *Campylobacter jejuni* into laboratory strains of *Escherichia coli*, giving the latter the ability to transfer glycans site-specifically onto acceptor proteins (Wacker et al., 2002). Following this seminal work, numerous additional heterologous glycosylation systems have been functionally reconstituted in *E. coli* (Feldman et al., 2005; Ihssen et al., 2010; Hug et al., 2011; Schwarz et al., 2011; Valderrama-Rincon et al., 2012; Shang et al., 2016; Keys et al., 2017; Tytgat et al., 2019), giving this simple organism the ability to produce a diverse array of complex glycomolecules. Hence, a more current definition of synthetic glycobiology is the purposeful alteration or rational construction of any glycosylation system using chemical and molecular biological approaches in conjunction with metabolic pathway engineering tools. Such synthetic systems have been instrumental in increasing our understanding of glycosylation networks and producing desired glycans and glycoconjugates.

## Synthetic Glycobiology Goes Cell-Free

While the majority of synthetic glycobiology efforts to date have involved living organisms, recent years have seen the emergence of cell-free systems as a new platform for synthetic glycobiologists to investigate and manipulate glycosylation outside of cells, leading to the birth of an entirely new field that we call cell-free synthetic glycobiology. Although still in its infancy, cell-free synthetic glycobiology has already helped to uncover the underlying mechanisms governing a variety of glycosylation reactions and enabled preparation of structurally-defined glycomolecules. The origins of this new field can be traced back almost 60 years ago when cell-free biology was used to decipher the genetic code (Nirenberg and Matthaei, 1961; Matthaei et al., 1962). Since that time, cell-free biology has matured into a well-established field in biological research (Carlson et al., 2011; Dudley et al., 2015; Silverman et al., 2020), undergoing a technological renaissance in the early twenty-first century (Shimizu et al., 2001; Jewett and Swartz, 2004; Jewett et al., 2008) that has catalyzed significant improvements in batch reaction yields (Caschera and Noireaux, 2014; Des Soye et al., 2019), operational volumes (Zawada et al., 2011; Yin et al., 2012), standardization of protocols (Kwon and Jewett, 2015; Kim et al., 2019), availability of various active lysate systems (Perez et al., 2016), incorporation of non-standard amino acids (Shimizu et al., 2005; Goerke and Swartz, 2009; Martin et al., 2018), and post-translational modifications (Oza et al., 2015; Jaroentomeechai et al., 2018) into biomolecule products. As a result, cell-free systems are now widely used, sometimes in tandem with cell-based systems, to produce complex biomolecules (Matthies et al., 2011), to prototype and optimize metabolic pathways (Karim and Jewett, 2016; Casini et al., 2018; Lim and Kim, 2019), for molecular sensing (Pardee et al., 2014, 2016), and to build and implement genetic networks (Takahashi et al., 2015; Swank et al., 2019). Owing to its open nature, cell-free reactions provide unprecedented flexibility to directly and precisely control compositions and conditions of a given system. Eliminating the biological membrane boundary also facilitates the integration of cell-free reactions with high-throughput screening tools (Su et al., 2016; Zhang et al., 2019), real-time monitoring, and automation (Georgi et al., 2016), resulting in significant reductions in design-build-test (DBT) timelines. Furthermore, the ability to harness cellular machineries without any impediments due to cell viability provides an opportunity to synthesize products and engineer biochemical pathways that otherwise exceed cellular toxicity tolerance (Kai et al., 2015; Thoring et al., 2017). Collectively, these versatile features are precisely what make cell-free platforms especially attractive for both mechanistic discovery and technological applications in glycoscience.

**Figure 1:**
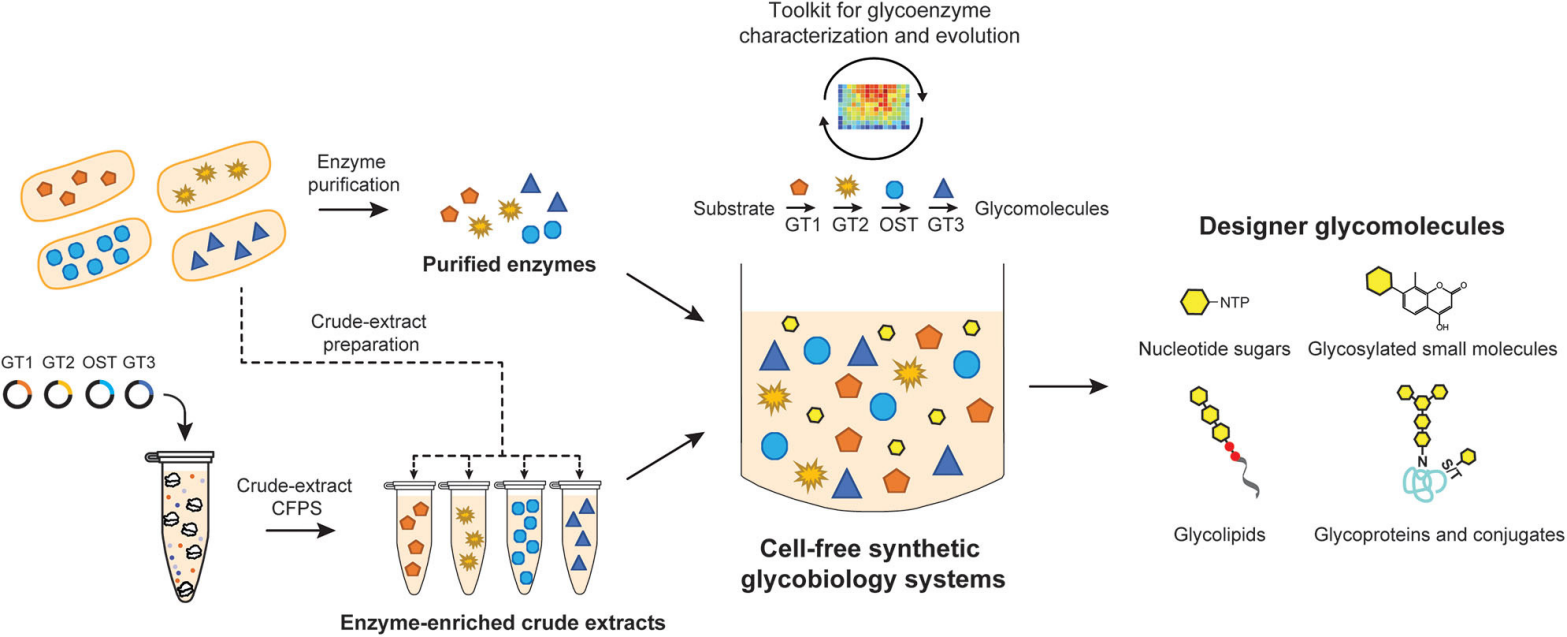
Cell-free synthetic glycobiology systems for on-demand biosynthesis of designer glycomolecules. Specific glycosylation reactions/pathways are assembled *in vitro* to synthesize designer glycomolecules including nucleotide sugar building blocks, small molecule glycosides, glycans, glycolipids, and glycoproteins. Glycoenzymes such as glycosyltransferases (GTs) and oligosaccharyltransferases (OSTs) can be prepared from living cells in a purified format or from cell-free protein synthesis (CFPS) expression platforms. Purified enzymes and/or enzyme-enriched crude lysates can then be combined in sequential reactions or in a single-pot to catalyze a prescribed glycosylation reaction. The open nature of cell-free systems allows each of the reaction components and their environment to be precisely controlled and monitored in real time, which in turn, facilitates the design-build-test cycle for an optimal output. Such open systems also provide great modularity as well as offer a unique possibility to integrate with high-throughput screening tools for constructing novel glycosylation pathways or evolving glycosylation enzymes.

In the sections that follow, we describe the utility of cell-free synthetic biology to produce structurally-defined glycomolecules including nucleotide-activated monosaccharide building blocks, glycosylated small molecules, free-reducing end glycans, glycolipids, and glycoproteins including conjugate vaccines. Characteristic features of these approaches are the synthesis of glycosylation components and the assembly of these components into functional glycosylation pathways that produce glycosylated molecules of interest in a well-controlled environment without the use of intact, living cells (Figure 1). In its simplest form, cell-free synthetic biology involves using purified enzymes to catalyze specific glycosylation reactions *in vitro* (Yu and Chen, 2016; Natarajan et al., 2018). Alternatively, to circumvent the time- and labor-intensive process of enzyme purification, *in situ* production of glycosylation enzymes in cell-free lysates has been used to assemble multi-step glycosylation reactions (Yu and Chen, 2016; Kightlinger et al., 2019). These biosynthesis-focused methods will be discussed in detail below, along with the use of cell-free synthetic biology as a tool to characterize and evolve glycosylation enzymes and pathways for designer functions. Collectively, these greatly simplified platforms offer exquisite control over reaction fluxes and compositions, which in turn become powerful tools to understand the biotransformation of glycomolecules. The primary focus here will be on cell-free biosynthesis approaches using glycosylation enzymes; detailed reviews on total chemical synthesis of glycans and glycomolecules can be found elsewhere (Ahmadipour and Miller, 2017; Krasnova and Wong, 2019).

## Cell-Free Enzymatic Synthesis of Nucleotide Sugar Building Blocks

Nucleotide sugars are essential to carbohydrate metabolism and glycomolecule biosynthesis in living organisms. These molecules consist of a nucleotide base linked to a monosaccharide *via* a mono- or pyrophosphate group. The attachment of nucleotidyl phosphate groups to monosaccharides is crucial for the recognition of sugar nucleotide-dependent (Leloir type) GTs, a major group of enzymes responsible for complex carbohydrate biosynthesis with high regio- and stereo-control. Nucleotide sugar biosynthesis has become a major focus area because the product outputs are indispensable for synthesizing complex glycoconjugates as well as for developing biochemical assays that enable characterization and engineering of glycosylation enzymes. Furthermore, these molecules and their derivatives have therapeutic potential as inhibitors of key enzymes in inflammation (Wang et al., 2018), cancer metastasis (Trapannone et al., 2016), and pathogen infection (Turnock and Ferguson, 2007).

**Figure 2:**
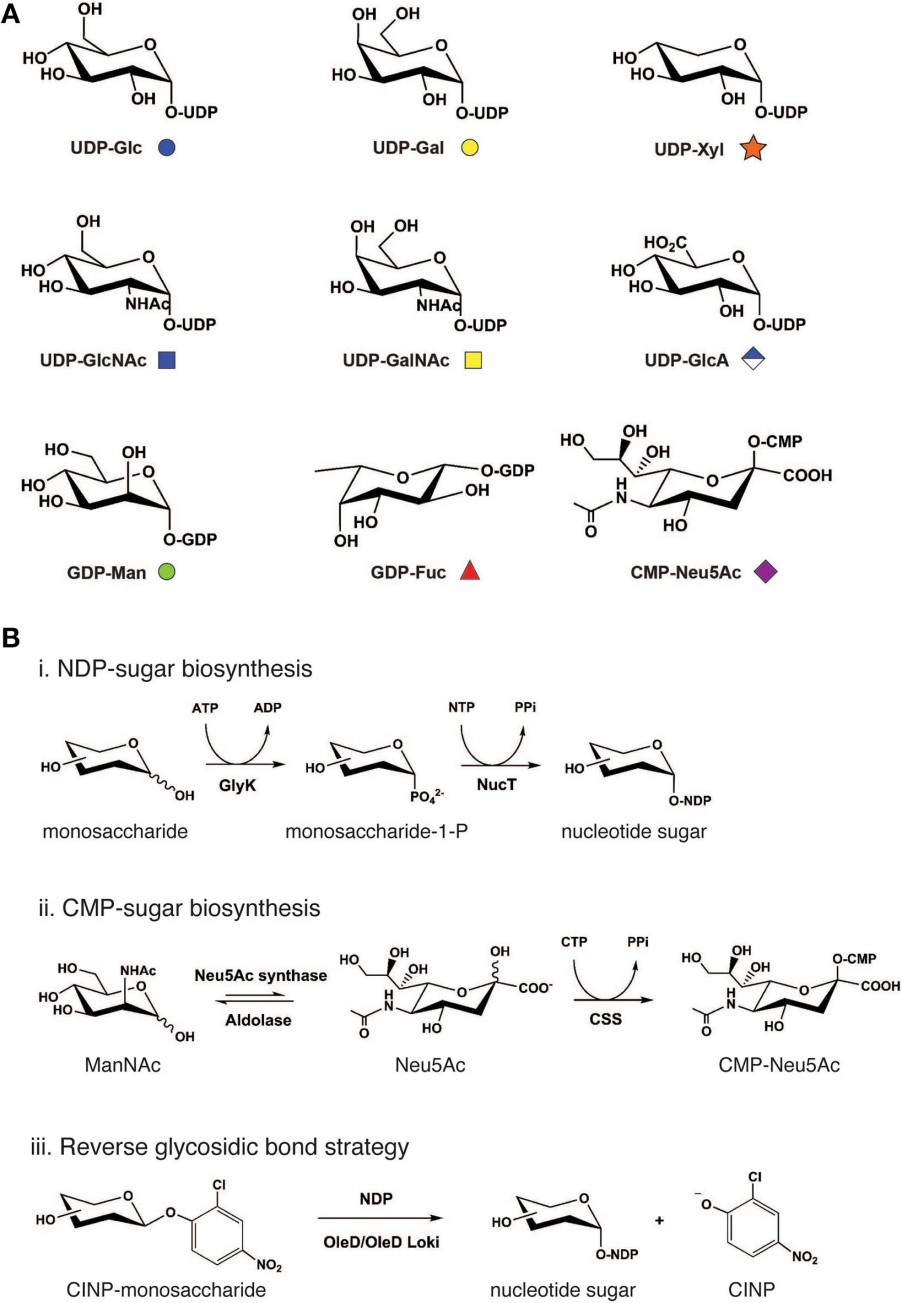
Cell-free synthetic glycobiology approaches to access monosaccharide building blocks. **(A)** Common mammalian nucleotide sugar structures along with its monosaccharide symbol nomenclature. **(B)** Cell-free bioenzymatic approaches to prepare sugar nucleotides. (Scheme i) Generally, NDP-sugars can be prepared from monosaccharides using a suitable glycokinase (GlyK), followed by nucleotidyltransferase (NucT). (Scheme ii) For the biosynthesis of CMP-Neu5Ac, ManNAc substrate is first converted to *N*-acetylneuraminic acid (Neu5Ac) using pyruvate and Neu5Ac synthase. Neu5Ac intermediate can then be phosphorylated by CMP-sialic acid synthetase to generate CMP-Neu5Ac. This synthetic scheme has also been successfully used to synthesize other CMP-sialic acids including *N*-glycolylneuraminic acid (Neu5Gc) and 2-keto-3-deoxy-d-glycero-d-galactonononic acid (Kdn) as well as their derivatives. (Scheme iii) Alternatively, reverse the glycosidic bond reaction utilizes aromatic glycosyl donors such as 2-chloro-4-nitrophenol (CINP) monosaccharide as a substrate in the reaction, which is converted to NDP-sugars by OleD/OleD Loki enzymes. Structures of nucleotide bases and phosphate groups are omitted for clarity. ATP, adenosine triphosphate; ADP, adenosine diphosphate; NTP, nucleoside triphosphate; PPi, inorganic pyrophosphate; and CTP, cytidine triphosphate.

Naturally occurring glycan repertoires are structurally complex. Yet despite this complexity, such structures in animal cells are generally diversified using only 9 common nucleotide-activated monosaccharide building blocks: UDP-Glc, UDP-Gal, UDP-Xyl, UDP-GlcNAc, UDP-GalNAc, UDP-GlcA, GDP-Man, GDP-Fuc, and CMP-Neu5Ac (Figure 2A). In nature, nucleotide-activated sugars are synthesized primarily from sugar-1-phosphate generated during glycolysis and, to a lesser extent, from salvage reactions whereby monosaccharides such as GalNAc, GlcNAc, Man, and Fuc are directly activated by nucleotide attachment (Cai, 2012). These general biosynthetic pathways are used as blueprints to design and construct nucleotide-activated sugars in cell-free systems.

To activate monosaccharides at the anomeric center by phosphorylation (Figure 2B, Scheme i), Nidetzky and co-workers devised a novel diastereoselective synthesis technique to prepare glucose 1-phosphate using sucrose phosphorylase and glucose 1-phosphatase (Wildberger et al., 2015). This method provides an economical route to prepare glucose 1-phosphate from simpler and cheaper inorganic phosphates, in comparison to traditional synthesis, which relies on the usage of nucleoside triphosphates (NTPs). In the past decade, several new sugar kinases have been characterized and utilized to prepare sugar 1-phosphates *in vitro* (Ahmadipour and Miller, 2017). The most recent example is *Leminorella grimontii* galactokinase (*Lg*GalK) reported by the Flitsch group (Huang et al., 2018). This enzyme was capable of phosphorylating C1 of galactose with high efficiency. Notably, *Lg*GalK displayed a broad substrate tolerance, including 3-deoxy-3-fluorogalactose and 4-deoxy-4-fluorogalactose, pointing to its potential use in the preparation of a library of nucleotide sugar analogs and their derivatives.

The utilization of completely enzyme-catalyzed synthesis of nucleotide sugars from simple monosaccharides in a single-pot reaction has become an attractive platform due to its simplicity, versatility, and ability to be coupled with GT-catalyzed reactions. One-pot multienzyme (OPME) synthesis technology was developed to realize this goal and has now become a widely used system (Li W. et al., 2019). In its simplest system, OPME involves modification of the monosaccharide with a nucleotide pytophosphate group using multiple enzymes in a single-pot reaction. These enzymes include a suitable glycokinase/nucleotidyltransferase to generate NDP-sugars, or a pair of Neu5Ac synthases/CMP-Neu5Ac synthethases to produce CMP-sialic acid (Figure 2B, Schemes i, ii). This simple one-pot reaction can then be coupled with other biosynthesis modules such as inorganic pyrophosphatase and GT reactions to construct a more elaborate carbohydrate structure on the aglycone molecule (Yu and Chen, 2016). Originally developed by the Chen group to prepare CMP-Neu5Ac (Yu et al., 2004) (Figure 2B, Scheme ii), state-of-the-art OPME technology is now capable of furnishing all common animal nucleotide sugars including UDP-Glc, UDP-Gal, UDP-GalNAc (Muthana et al., 2012), UDP-GlcNAc (Chen et al., 2011), UDP-GlcA (Muthana et al., 2015), UDP-Xyl (Errey et al., 2004), and GDP-Man (Li et al., 2013). In addition to natural sugars, the open nature of cell-free OPME system facilitates the synthesis of many non-natural sugars, for example: NDP-Man (Mizanur and Pohl, 2009); UDP-CH_2_-Gal*p* (Partha et al., 2010); UDP-Glc-6-deoxy-6-F (Caputi et al., 2013); 5-position base-modified sugar nucleotides (Wagstaff et al., 2015); and UDP-4-F-GlcNAc (Schultz et al., 2017). These nucleotide sugar analogs are not only useful as mechanistic probes and biochemical reporters, but they also could find use as novel enzyme inhibitors.

An emerging approach for the biosynthesis of nucleotide sugars is the reverse glycosidic bond strategy (Figure 2B, Scheme iii), which was first described by Thorson and co-workers (Zhang et al., 2006; Gantt et al., 2011). Breaking glycosidic bonds to form nucleotide sugars is thermodynamically unfavorable as it produces highly energetic molecules from a relatively stable covalent bond between the carbohydrate and aglycone. To overcome this thermodynamic barrier, the authors designed a series of aromatic sugar donors and successfully utilized these glycoside donors to shift the equilibria toward glycosidic bond breakage in a reaction catalyzed by a macrolide-inactivated GT mutant, namely the OleD variant TDP-16. The enzyme was further evolved by the same group to a Loki variant that is capable of recognizing a broader set of sugar donors and NDP acceptors (Gantt et al., 2013). Taken together, the development of both the OPME platform and reverse glycosidic bond approach have provided an ever-expanding library of nucleotide sugars that can be used to assemble more elaborate glycomolecules for fundamental studies and applications in glycoscience.

## Cell-Free Biosynthesis of Glycosylated Small Molecules

The past decade has seen the emergence of cell-free systems, both in the form of purified enzymes and enzyme-enriched crude extracts, as a platform to supply high-value and commodity chemicals. By sidestepping the use of live-cell factories, cell-free systems enable biosynthesis schemes in which all resources can be focused toward preparation of the desired product while at the same time allowing a wider range of precisely-controllable operational conditions (Dudley et al., 2015). More importantly, liberation from cell viability allows cell-free reactions to synthesize target molecules at concentrations that exceed the cellular toxicity limit (Swartz, 2018). Together, these features have made cell-free systems an attractive platform for high-yield biomanufacturing of target compounds as well as for prototyping novel biosynthetic routes to their production. To date, several metabolic pathways and enzyme cascades have been implemented in cell-free formats including, but not limited to, those synthesizing antibiotics (Kim et al., 2000), cannabinoid precursors (Valliere et al., 2019), commodity alcohols (Guterl et al., 2012; Kay and Jewett, 2015, 2020), food-grade antimicrobials (Kawai et al., 2003), glycolysis intermediates (Bogorad et al., 2013), hydrogen gas fuel (Martin Del Campo et al., 2013), isoprene compounds (Korman et al., 2014; Dudley et al., 2016), natural products (Goering et al., 2017), and nucleotides (Schultheisz et al., 2008, 2011).

Natural products and their derivatives including flavonoids, alkaloids, polyphenols, terpenoids, antibiotics, vitamins, and sweeteners are a major group of high-value chemicals with utility as anti-cancer, anti-inflammatory, antioxidant, and antibacterial agents. However, their clinical evaluation and utility are often limited by poor solubility, low stability, and, severe toxicity resulting from their inherent structural properties. Modifying such chemicals with a carbohydrate moiety to form the *O*-, *N*-, *S*-, or *C*-linked glycosides is a universal way to circumvent these limitations (Desmet et al., 2012). These glycosylation reactions are generally mediated by Leloir-type GTs with different types of glycosyl donors. For example, OleD from *Streptomyces antibiotics* and YjiC from various *Bacillus* species are extensively used for cell-free glycosylation of small molecules. Both enzymes accept a diverse set of NDP-sugars as glycosyl donors (Gantt et al., 2013) and show promiscuous substrate specificity (Zhou et al., 2013). The Thorson group conducted a pilot-scale, cell-free reaction in which purified OleD was shown to glycosylate more than 100 small molecules covering various classes of natural products including flavonoids, alkaloids, antibiotics, steroids, and stilbenes (Zhou et al., 2013). A similar study by Sohng and coworkers revealed that purified YjiC from *Bacillus licheniformis* can glucosylate more than 23 structurally diverse flavonoids with high (~80–100% conversion) efficiency in a cell-free reaction (Pandey et al., 2014). YjiC from a related species, *Bacillus subtilis 168*, was also capable of *in vitro* glucosylation of numerous drug-like molecular scaffolds including 19 diverse structures of flavonoids, phenylketones, curcuminoids, lignins, triterpenes, anthraquinone, stilbene, zingerone, and aromatic aglycones with nucleophilic groups (Dai et al., 2017). Notably, both OleD (Gantt et al., 2008) and YjiC (Dai et al., 2017) are multi-functional GTs that can catalyze the formation of *O*-, *N*-, and *S*-glycosidic linkages. Along similar lines, Walsh and coworkers reported cell-free *C*-linked glycosylation using purified *C*-glycosyltransferase IroB from uropathogenic *E. coli* strain CFT073 that was capable of decorating enterobactin substrates with several glucose molecules (Fischbach et al., 2005).

While much attention has been given to the GT-mediated small molecules glycosylation using nucleotide-activated sugars, alternative biosynthetic routes using either glycoside phosphorylases (GPs) or glycosyl hydrolases (GHs) should not be overlooked. GP-catalyzed reactions can achieve rigid stereo- and regio-selective synthesis from a relatively stable and economically-viable sugar phosphate substrate, making this synthetic route especially attractive for large-scale synthesis (Nakai et al., 2013). By using purified sucrose phosphorylase and cellobiose phosphorylase with sucrose as glycan donors, Desmet and colleagues were able to prepare a series of structurally diverse glycosylated phenolic compounds, albeit at relatively low yields compared to GT-driven reactions (De Winter et al., 2015). The resulting glycosides exhibited significantly improved solubility and thermal stability, although their antioxidant activities were decreased to different extents. Alternatively, non-activated sugars (e.g., sucrose, starch) can also be used as glycan donors for the GH enzyme to glycosylate small molecules. Many GH enzymes exhibit dual functionalities and are thus capable of catalyzing both hydrolysis (glycosidic bond breakage) and transglycosylation (glycosidic bond formation) reactions. Whereas, GHs generally catalyze glycoside hydrolysis *in vivo*, the equilibrium of their reactions can be effectively reversed *in vitro* under certain conditions, making these enzymes suitable for cell-free transglycosylation reactions (Mladenoska, 2016). One representative study utilized a cyclodextrin glucanotransferase from *Thermoanaerobacter* sp. to transfer a glucosyl group from starch to pterostilbene *in vitro* (Gonzalez-Alfonso et al., 2018). Although glycosylation was found to slightly reduce its antioxidant activity, the resulting pterostilbene α-D-glucopyranoside exhibited improved solubility and reduced toxicity.

## Enzyme-Mediated *in vitro* Technologies For Assembling Glycans and Glycolipids

Complex carbohydrates or glycans in their unconjugated form (free glycans) are valuable reagents, finding use in both fundamental research and biomedical applications. An outstanding example is the use of structurally-defined free glycans to construct glycoarrays that enable high-throughput screening of molecular interactions between glycan epitopes and carbohydrate-binding entities including proteins and even whole organisms (Rillahan and Paulson, 2011). Since their first report (Fukui et al., 2002; Blixt et al., 2004), glycoarrays have proven to be tremendously useful for the discovery of antibodies, lectins, and immune receptors against carbohydrate antigens as well as for determining the substrate-specificity of various GTs (Blixt et al., 2008; Wen et al., 2018). To date, the Consortium for Functional Glycomics (CFG) and the Glycosciences Laboratory at Imperial College have developed two of the largest glycoarray libraries, respectively, consisting of ~609 mammalian glycans (Mcquillan et al., 2019) and ~796 neoglycolipid glycan structures (Palma et al., 2014; Li and Feizi, 2018). Another important application of free glycans is in the synthesis and development of conjugate vaccines, which are particularly effective against various bacterial pathogens (Moeller et al., 2018).

One of the major impediments to using free glycans as described above is accessibility of pure glycans at sufficient quantities. Initially, glycan libraries were obtained from natural sources such as microbes, plants, or animal products. In this process, glycans were separated from their bioconjugates through chemical or enzymatic hydrolysis followed by tedious, multistep purifications (Rillahan and Paulson, 2011). However, the high diversity of glycan structures present in natural samples makes it very difficult to acquire highly pure compounds using this approach. An alternative to harvesting glycans from natural sources is the use of chemical synthesis methods to generate free glycans from simple monosaccharide precursors. Chemical synthesis typically involves performing iterative rounds of glycosylation reactions utilizing a protecting group scheme that enables functionalization of a single hydroxyl group for sugar attachment. However, such *de novo* synthesis requires lengthy organic chemistry procedures, often necessitating highly specialized individuals and instrumentation. Some of these limitations have been alleviated by the introduction of automated solid-phase oligosaccharide synthesizers for the rapid synthesis of glycans as described by Seeberger and coworkers (Plante et al., 2001). By adopting solid-phase synthesis, excess amounts of glycosyl donor can be used to drive reactions to completion and the removal of unwanted side products or reagents can be done in a single wash step. Since the time of its inception, the technology has now matured into a fully commercial system known as Glyconeer 2.1 (Hahm et al., 2017). These developments notwithstanding, the chemical synthesis of glycans remains a significant challenge due to the complexity in achieving stereo- and regio-selective synthesis. Selecting appropriate protective groups to achieve the desired glycosidic linkage remains one of the main hurdles and becomes more difficult as the complexity of the glycan architecture increases.

To circumvent the need for protecting group manipulation, the development of cell-free glycan synthesis systems that leverage enzymes such as GTs, GHs, and other glycan-processing enzymes is an attractive alternative. Enzymatic glycosylation permits precise stereo- and regio-controlled synthesis with high conversions using unprotected monosaccharides as substrates. Reactions generally proceed under mild, aqueous conditions without the need for toxic and harsh organic reagents. Using bio- and/or chemoenzymatic synthesis tools, several natural and engineered glycan libraries have recently been constructed including asymmetric multi-antennary *N*-glycans (Wang Z. et al., 2013), glycosphingolipid glycans (Yu et al., 2016), authentic human type *N*-glycans (Li L. et al., 2015; Hamilton et al., 2017), *O*-mannosyl glycans (Meng et al., 2018; Wang S. et al., 2018), human milk oligosaccharides (HMOs) (Xiao et al., 2016; Prudden et al., 2017), and tumor-associated antigens (Li P. J. et al., 2019; 'T Hart et al., 2019). Similar strategies have been adopted for cell-free enzymatic synthesis of glycolipid libraries including those from bacterial (Glover K. et al., 2005), animal (Stubs et al., 2010), and human origins (Li S. T. et al., 2019). Many of these glycan and glycolipid libraries have been employed to construct glycan microarrays for profiling glycan-binding molecules such as lectins and antibodies as well as for gaining mechanistic insights into glycosylation reactions.

Cell-free enzymatic glycan synthesis can also be integrated with automated systems for more expeditious glycan assembly. To achieve this goal, several developments that simplify purification processes and increase conversion efficiencies have recently been reported. For example, the Linhardt group demonstrated the use of a fluorous tag to capture heparin sulfate products directly from solution (Cai et al., 2014). Additional advances include a photocleavable linker that enables chemoenzymatic synthesis of tumor-associated glycan epitopes (Bello et al., 2015) and an ion-exchange purification technique that aids in cell-free biosynthesis of HMOs (Zhu et al., 2017). These advances, along with many others, have been instrumental in realizing the goal of a fully automated enzymatic glycan synthesizer, several of which have now been reported or are in late stages of development. For example, Nishimura and coworkers developed an artificial “Golgi apparatus” to prepare sialyl Lewis X derivatives using a dendrimer-based solid support (Matsushita et al., 2010). Their process took 4 days and provided an overall yield of 16%. One of the main challenges of the Golgi apparatus was that it required multiple filtration-purification steps that hindered its efficiency. To address these challenges, Wang and colleagues recently combined a thermosensitive polymer with a commercially available peptide synthesizer to mediate automated glycan assembly (Zhang et al., 2018). Their system was able to prepare several blood group antigens and ganglioside glycans with yields ranging from 27 to 38% within 1–2 days. Coincidentally, Boons and coworkers simultaneously developed a similar automated system using a set of water-soluble sulfate tags for a catch-and-release synthesis strategy (Li T. et al., 2019). The sulfate tags were compatible with a range of glycosylation enzymes and, more importantly, were readily adapted to a custom-designed automated glycosynthesizer. Using this fully automated platform, quantitative amounts of complex glycans including gangliosides, HMOs, poly-*N*-acetyllactosamine (poly-LacNAc) derivatives, and *N*-glycans could be prepared in a less labor- and time-intensive process. Despite the small number of examples, the use of automated enzymatic glycan synthesis platforms shows significant promise, both as stand-alone systems and in combination with automated chemical synthesis (Fair et al., 2015). With rapid developments in automation, instrumentation, solid-support matrices, reliable tags and linkers, as well as a growing collection of accessible glycosylation enzymes, a fully mature and reliable enzymatic glycan synthesizer capable of synthesizing virtually any complex carbohydrate structure appears to be within reach.

## Cell-Based and Cell-free Biosynthesis of Structurally-Defined Glycoproteins

Protein glycosylation, the covalent attachment of glycans onto specific amino acid residues within a polypeptide chain, is one of the most common post-translational modifications in nature (Apweiler et al., 1999; Khoury et al., 2011). The attached glycan can significantly affect the intrinsic properties of its recipient protein such as folding, stability, localization, antigenicity, and immunogenicity (Helenius and Aebi, 2001; Shental-Bechor and Levy, 2009; Skropeta, 2009). Aberrant protein glycosylation is widely linked to disease states such as cancer and autoimmune diseases (Ohtsubo and Marth, 2006; Peixoto et al., 2019). Furthermore, the majority of therapeutic proteins including monoclonal antibodies are glycosylated and the manner of glycosylation often determines protein drug stability and biological function (Jefferis, 2009a).

More than 40 different types of carbohydrate-to-protein linkages have been identified to date. Among these, glycan installation at the asparagine (*N*-linked) and serine/threonine (*O*-linked) residues constitutes the greatest proportion of glycoproteins (Spiro, 2002). Protein glycosylation is highly dynamic and the glycan profile is controlled both spatially and temporally by the amino acid sequence, the local structural conformation of the glycosylation site, and the expression level of glycoenzymes at different stages of cellular development (Colley et al., 2015). Thus, glycoproteins are generally found in nature as a mixture of glycoforms sharing the same protein backbone but a variety of glycan structures. This intrinsic heterogeneity makes it challenging to decipher how specific glycoforms impact the structure and function of a modified protein. It has also been proven to be a major impediment for the development of glycoprotein-based therapeutics as the consistent ratio and identity of glycoforms are essential for reproducible clinical efficacy and safety of the biologic (Wang and Lomino, 2012). To address these challenges, a variety of glycoengineering approaches have been reported that involve the design and construction of molecular, cellular, and whole-organism systems with tunable glycosylation. In the following sections, we describe recent progress in cellular glycoengineering as well as highlight emerging cell-free technologies that leverage diverse glycoenzymes to produce structurally-defined glycoproteins for a range of downstream applications.

### Cell-Based Glycoengineering

**Figure 3:**
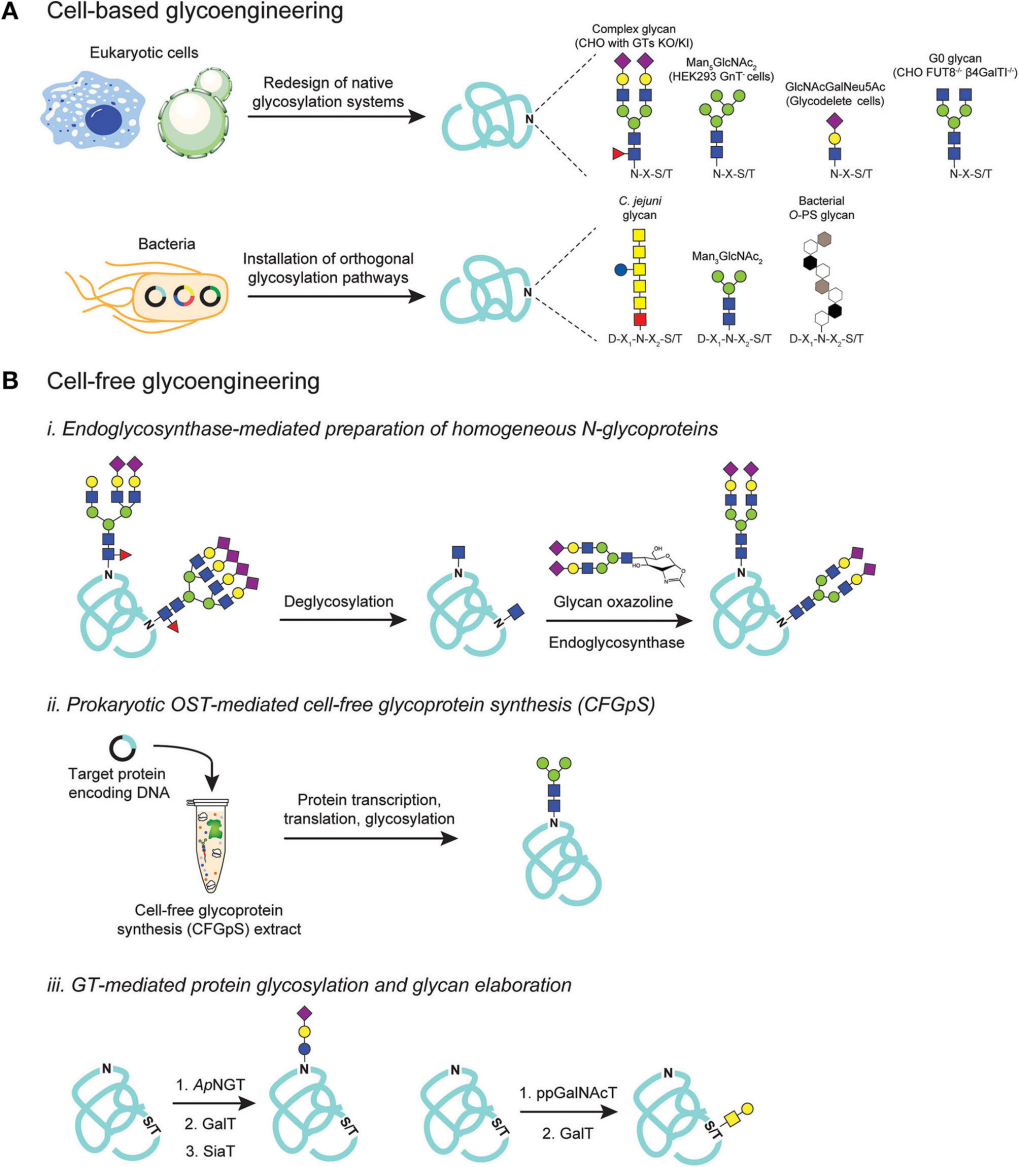
Glycoengineering strategies for producing homogeneous glycoproteins. **(A)** Cell-based glycoengineering involves redesign of native protein glycosylation pathways in the host organism to control glycoforms. Precise genome editing strategies such as ZFNs and CRISPR/Cas9 systems have been employed to knock-out (KO) and knock-in (KI) genes to alter endogenous glycosylation networks for producing proteins bearing desirable glycoforms such as Man_5_GlcNAc_2_, GlcNAcGalNeu_5_Ac, G0, and complex-types with sialic acid-capped glycoforms. Alternatively, orthogonal expression of protein glycosylation pathways in *E. coli* can be utilized to produce proteins that are site-specifically modified with bacterial *N*-glycans, eukaryotic glycans such as Man_3_GlcNAc_2_, and bacterial *O*-polysaccharide (*O*-PS) antigens. **(B)** Cell-free glycoengineering using glycoenzymes including (i) endoglycosynthases (ENGases), (ii) prokaryotic oligosaccharyltransferases (OSTs), and (iii) glycosyltransferases (GTs). For ENGase-mediated glycosylation, glycoproteins bearing heterogenous *N*-glycoforms are deglycosylated using specific glycosyl hydrolases (GHs) to generate monosaccharide handle such as GlcNAc at the native glycosylation site. Pre-synthesized glycan structures containing an oxazoline functional group at the reducing end are then used as glycosyl donor in a reaction catalyzed by ENGase to remodel glycans to homogeneity. For prokaryotic OST-mediated glycosylation, cell-free extract is generated from glycoengineered *E. coli* such that the extract is enriched with all necessary gene transcription, protein translation, and protein glycosylation machineries. Supplementing extracts with DNA encoding target protein co-activates protein synthesis and site-specific protein glycosylation. For GT-mediated protein glycosylation, sequential glycosylation reactions are carried out, beginning with installation of an initial monosaccharide on the protein using a specific GT such as *Ap*NGT that installs Glc on asparagine residues and ppGalNAcT that modifies serines or threonines with GalNAc. The monosaccharide primer can then be extended, directly on glycoprotein, by a series of specific GTs such as GalT and SiaT to generate a final glycoform.

There is a long history of cellular glycoengineering in eukaryotes including in mammalian cells, plants, and yeasts (Bertozzi et al., 2009). Among these, the glycoengineering of Chinese hamster ovary (CHO) cultures has dominated the field as it is still the most commonly used host cell line in the biopharmaceutical industry (Walsh, 2018). Many groups have explored glycosylation control using genetic manipulation to overexpress genes encoding glycoenzymes such as Golgi-resident GTs (Weikert et al., 1999; Son et al., 2011) (Figure 3A). Small molecule inhibitors targeting glycoenzymes such as kifunensine (Elbein et al., 1990) and swainsonine (Elbein et al., 1981) have also been successfully used to regulate a protein's glycoform in CHO culture (Ehret et al., 2019). More recently, systems biology and bioinformatics tools have been used to model glycosylation reaction networks in order to explore and quantify how perturbations to glycosylation parameters affect the cell (Neelamegham and Liu, 2011). Coupling this insight with precise genome editing tools will offer unprecedented freedom to glycoengineer organisms with greater control over glycoprotein products. A landmark achievement in this regard was reported by the Clausen group whereby quantitative genomics data and precise genome editing was used to generate a panel of CHO cells with specific GT gene knock-outs (Yang et al., 2015). These glycoengineered CHO cells were used to screen and identify GT genes that play a major role in regulating protein *N*-glycosylation within the CHO cell glycome. Such knowledge, in turn, provided a blueprint for genetic reconstruction of CHO cells with desirable glycosylation capacities including those producing human-like α2,6-linked sialic acid-capped glycoforms on therapeutic proteins such as human IgG and Erythropoietin (EPO) (Yang et al., 2015; Caval et al., 2018; Schulz et al., 2018). Another notable example from the Weiss group explored the use of CRISPR/Cas9 to implement synthetic gene circuits in CHO cells, allowing tunable *N*-glycan profiles of CHO culture-derived IgGs in a small molecule concentration-dependent manner (Chang et al., 2019). More recently, precision gene editing was used to create a library of validated CRISPR/Cas9 guide RNA targeting constructs for all human GT genes (Narimatsu et al., 2018). This gRNA library was subsequently applied to create an array of HEK293 cells displaying the human glycome (Narimatsu et al., 2019). This cell-based library of human glycan structures should become a valuable resource for dissecting glycan biosynthesis and glycomolecule interactions within a native physiological context (Narimatsu et al., 2019). It should also be pointed out that advances in cell-based glycoengineering have extended beyond mammalian cells, with significant progress toward producing homogeneous glycoforms in other eukaryotes including yeast (Hamilton et al., 2003; Wildt and Gerngross, 2005), microalgae (Barolo et al., 2020), insect cells (Toth et al., 2014), and plant cell cultures (Montero-Morales and Steinkellner, 2018; Hurtado et al., 2020). Comprehensive reviews of the glycoengineering approaches developed in these eukaryotic systems have been published elsewhere (Hamilton and Zha, 2015; Heffner et al., 2018).

Not to be outdone, glycoengineering in prokaryotes has emerged as an attractive strategy for cell-based production of homogenous glycoproteins (Figure 3A). The discovery of a *bona fide N*-linked protein glycosylation pathway in the mucosal bacterium *C. jejuni* (Szymanski et al., 1999; Gross et al., 2008), and its functional reconstitution in *E. coli* (Wacker et al., 2002), laid the foundation for the development of a bacterial glycoengineering system. Owing to its lack of any native protein glycosylation systems, *E. coli* offers a blank canvas on which prescribed, orthogonal glycosylation pathways can be assembled without concern over interference from endogenous glycoenzymes. Combined with its fast growth, ease of genetic manipulation, and the ability to express a wide range of recombinant proteins, *E. coli* cells equipped with glycosylation machinery are capable of biosynthesizing designer glycoproteins bearing various therapeutically-important glycan epitopes such as the eukaryotic core *N*-glycan Man_3_GlcNAc_2_ (Valderrama-Rincon et al., 2012; Glasscock et al., 2018), bacterial *O*-polysaccharide (O-PS) antigen structures (Feldman et al., 2005), human blood group antigens (Hug et al., 2011; Shang et al., 2016), authentic human *O*-glycans (Du et al., 2018; Natarajan et al., 2020), and polysialic acid-containing glycans (Keys et al., 2017; Tytgat et al., 2019). Taken together, efforts in cellular glycoengineering have yielded a variety of expression platforms, both prokaryotic, and eukaryotic, for producing glycoproteins with chemically-defined carbohydrate structures. Further improvement of the existing methods as well as the invention of entirely new technologies are anticipated to expand the glycoprotein expression toolkit available to scientists and engineers.

### Cell-Free Glycoengineering

While glycoengineering in living cells is offering novel engineered organisms with desirable glycosylation capacities (Steentoft et al., 2014; Tejwani et al., 2018), the repertoire of accessible glycan structures remains limited. Moreover, genetic manipulation of host cells is often non-trivial as it is constrained by a multitude of factors such as the essential nature of cellular glycosylation and its impact on cell viability, difficulties in precisely tuning the expression of glycosylation components, and intracellular complexity especially with respect to the plethora of native GTs that compete for glycosylation substrates and catalyze the formation of unwanted glycoforms. On the other hand, cell-free approaches are not restricted by these cellular limitations and can provide more stringent control over glycan assembly and installation reactions to obtain highly pure, structurally-defined glycoproteins. Many of the early efforts in cell-free glycoengineering focused on total synthesis of glycoproteins using native chemical ligation and/or chemoselective ligation (Wang and Davis, 2013), and significant progress on this front has been made as documented in reports describing the assembly of large and complex glycoproteins including the α- and β-subunits of human hormone (Aussedat et al., 2012; Nagorny et al., 2012), interferon (Sakamoto et al., 2012), RNase C (Piontek et al., 2009a,b), and human erythropoietin (Wang P. et al., 2013). In parallel, enzyme-mediated cell-free glycoprotein synthesis is emerging as a tool to complement chemical methods for synthesizing homogeneous glycoproteins. As mentioned earlier, enzymatic glycosylation offers precise control over stereo- and regio-chemistry without the need for chemical protecting groups, making it especially attractive for preparative-scale biosynthesis of complex glycoproteins. In the text that follows, we present three major cell-free enzymatic approaches for preparing glycan-defined glycoproteins.

#### Endoglycosynthase-Mediated Preparation of Homogeneous N-Glycoproteins

GHs are a class of glycoenzymes responsible for breaking specific glycosidic bonds in glycomolecules. They exhibit dual functionalities depending on whether a water molecule (hydrolysis reaction) or an activated –OH group of another carbohydrate acceptor (transglycosylation reaction) attacks an enzyme-substrate complex during catalysis (Li and Wang, 2018). The latter activity has pointed to the potential use of GHs in preparing glycoproteins by the *en bloc* transfer of pre-synthesized glycans from a glycosyl donor onto an acceptor protein (Figure 3B, Scheme i). The most commonly used acceptor is a protein containing a single GlcNAc moiety, which can be generated *via* chemical synthesis or by enzymatic glycan trimming of glycoproteins derived from mammalian (Goodfellow et al., 2012; Giddens et al., 2016), yeast (Liu et al., 2018), or microbial cultures (Schwarz et al., 2010).

Following initial attempts to use transglycosylation to synthesize glycosides (Kobayashi et al., 1991), two seminal discoveries have significantly propelled progress in the field: (i) the generation of GH mutants called glycosynthases that favor transglycosylation over hydrolysis (Mackenzie et al., 1998); and (ii) the use of sugar oxazolines as glycosyl donors for glycosynthases, which dramatically improves transglycosylation yields (Umekawa et al., 2008). To date, more than a dozen GHs including β-glycosidases (GH 1), α-galactosidases (GH 35, 36), α-fucosidases (GH 29), and endohexosaminidases (GH 18, 20, 25, 56, 84, and 85) have been cataloged and transformed into glycosynthases (Danby and Withers, 2016). Among these, endo-β*-N-*acetylglucosaminidases (ENGases) such as those isolated from *Arthrobacter protophorminae* (EndoA), *Mucor hiemalis* (EndoM), *Streptococcus species* (EndoD, S, and H), and *Elizabethkinga meningoseptica* (EndoF3) of the GH 18 and 85 families, have attracted the greatest attention due to their ability to cleave between the chitobiose core of *N*-glycans (Barreaud et al., 1995). A series of ENGase-mutants with improved transglycosylation activity has been isolated and successfully used for convergent synthesis and glycan remodeling of diverse *N*-glycoproteins including Saposin C glycopeptide with a complex-type *N*-nona-saccharide (Hojo et al., 2012), RNase B bearing a high mannose-type *N*-glycan (Takegawa et al., 1995; Amin et al., 2011), glycosylated insulin (Tomabechi et al., 2010), glycosylated HIV peptide antigen (Amin et al., 2013), fibrinogen (Giddens et al., 2016), and, most notably, IgG-Fc with a homogenous glycoform (Fan et al., 2012).

Monoclonal antibodies (mAbs) continue to be one of the fastest growing classes of biotherapeutics (Walsh, 2018). All therapeutic mAbs contain an *N*-glycan at the conserved N297 residue within the Fc region. The impact of Fc glycan on the physicochemical properties and effector functions of mAbs has been well-documented (Jefferis, 2009b). Thus, the ability to obtain mAbs in a pure glycoform not only guarantees a reproducible route for producing safe biologics, but also opens the door for engineering more effective therapeutic mAbs. With this in mind, Lai-Xi Wang and coworkers isolated two EndoS mutants that could effectively remodel the glycan of an intact IgG (Huang et al., 2012). The utility of their approach was demonstrated through the remodeling of glycans on the therapeutic mAb, Rituximab, resulting in well-defined glycoforms including Man_3_GlcNAc_2_ (M3) azide-containing M3, Gal_2_GlcNAc_2_Man_3_GlcNAc_2_ (G2), and NeuNAc_2_Gal_2_GlcNAc_2_Man_3_GlcNAc_2_Fuc (G2FS2). The glycan remodeling reactions were efficient, yielding sufficient quantities of each glycoform to be examined for binding affinity with Fcγ receptors. Following this breakthrough, ENGase-mediated chemoenzymatic glycan remodeling has become widely adopted by many research groups to generate homogeneous glycoforms of therapeutic mAbs, including Rituximab (Lin et al., 2015) and Herceptin (Kurogochi et al., 2015; Liu et al., 2018), with a relatively large glycoform library. Recent work from the Davis group further improved the transglycosylation reaction conditions with reduced unfavorable side reactions such as chemical glycation. The most optimal reaction yielded the purest glycoform of Herceptin (~90%) to date (Parsons et al., 2016). Finally, the utility of chemoenzymatic transglycosylation was recently extended to install phosphorylated glycans (Priyanka et al., 2016) and to remodel *N-*linked glycans in the Fab region (Giddens et al., 2018). Importantly, the ability to generate relatively homogenous mAb glycoforms is providing insights into how specific carbohydrate epitopes modulate conformational changes and effector functions of antibodies, including antibody-dependent cellular cytotoxicity (ADCC), complement-dependent cytotoxicity (CDC), and anti-inflammatory activities.

#### Prokaryotic OST-Mediated Cell-Free Glycoproteins Biosynthesis

The ability to reconstitute glycoprotein biosynthesis in a well-defined, cell-free environment has the potential to transform the study of glycoscience. In such a system, not only can a particular step in glycan assembly, glycan modification, and glycan installation on the protein be carefully interrogated, but it can also facilitate the construction of engineered glycosylation pathways for making specific glycoforms of a protein. Such systems are inspired by and borrow components from natural glycosylation mechanisms found in eukaryotes, and more recently in prokaryotes.

In eukaryotes, *N*-glycoprotein biosynthesis involves the transfer of a preassembled glycan (Glc_3_Man_9_GlcNAc_2_) from a dolichyl-pyrophosphate carrier to an asparagine residue within the Asn-Xaa-Thr/Ser (where X ≠ Pro) consensus sequon of a nascent polypeptide chain by an oligosaccharyltransferase (OST) enzyme (Aebi, 2013). The precursor *N*-glycan on glycoproteins then undergoes a series of GH-mediated glycan trimming and GT-mediated glycan elaboration steps in the ER and Golgi to yield the final glycoform of the protein (Berger, 1985; Arigoni-Affolter et al., 2019). The OST is a key enzyme of this pathway and consists of a protein complex containing multiple transmembrane subunit proteins, including the catalytic subunit STT3 (Kelleher and Gilmore, 2006). Early work from the Coward and Imperiali groups devised *in vitro* glycosylation assays to gain mechanistic understanding of the substrate specificity and activity of the yeast OST (Xu and Coward, 1997; Tai and Imperiali, 2001). Many of these studies were done using crude extract-containing detergent-solubilized OSTs from yeast microsomes to catalyze glycan transfer from dolichyl lipid-linked oligosaccharides (LLOs) onto peptide acceptors containing a glycosylation motif (Sharma et al., 1981; Srinivasan and Coward, 2002). Due to the inherent structural complexity, the preparation of membrane-bound OST complexes has proven difficult, often leading to inactive or unstable enzymes. Recent advances in biochemical techniques, however, have now made it possible to obtain highly-pure and active OST complexes, including those from humans, for *in vitro* functional characterization and most importantly structural elucidation (Bai et al., 2018; Wild et al., 2018; Ramirez et al., 2019). Nevertheless, the preparation of glycoproteins by eukaryotic OST-mediated *in vitro* glycosylation has yet to be realized. Key impediments include the inaccessibility of dolichyl LLO libraries (Gibbs and Coward, 1999) and the uncertainty of whether eukaryotic OSTs, which operate co-translationally, can also post-translationally modify target proteins. To date, *in vitro* glycosylation reactions catalyzed by eukaryotic OSTs have only been performed with short synthetic peptide acceptors and it remains to be seen whether these enzymes can efficiently glycosylate fully folded-proteins *in vitro* (Bai et al., 2018; Wild et al., 2018; Ramirez et al., 2019).

Similar to eukaryotes, *N*-linked protein glycosylation in certain Proteobacteria such as *Campylobacter* and *Helicobacter* species involves *en bloc* transfer of glycans from undecaprenyl-pyrophosphate (Und-PP) glycolipids onto conserved glycosylation motifs within the protein chain (Szymanski and Wren, 2005; Nothaft and Szymanski, 2010). Bacterial OSTs share a conserved architecture with eukaryotic STT3s with the exception that bacterial OSTs are single-subunit enzymes (Szymanski et al., 1999; Dell et al., 2010). Shortly after the discovery of the first *bona fide N-*glycosylation system in *C. jejuni*, Aebi and colleagues demonstrated the functional transfer of the *C. jejuni* protein glycosylation locus (*pgl*) into *E. coli* (Wacker et al., 2002), which not only facilitated mechanistic studies of the pathway but opened the door to bacterial glycoengineering.

By leveraging glycoengineered strains of *E. coli*, early work demonstrated that the *C. jejuni* OST (hereafter *Cj*OST) has a more stringent substrate specificity than eukaryotic OSTs, requiring an extended glycosylation sequon, Asp/Glu-X_−1_-Asn-X_+1_-Ser/Thr (where X_−1_, X_+1_ ≠ Pro) (Kowarik et al., 2006b). The so called “minus-two rule” of the *Cj*OST, requiring an acidic amino acid residue at the −2 position of the glycosylation site, did not strictly apply to other bacteria, as several *Cj*OST homologs, such as those found in *Desulfovibrio, Helicobacter*, and deep sea vent bacterial species, were observed to have significantly relaxed substrate specificity (Ollis et al., 2015; Mills et al., 2016). Regardless of their specific sequon preferences, these enzymes are capable of installing glycans onto sequons that have been engineered at the N- and C-termini and in flexible regions of heterologous proteins (Fisher et al., 2011; Lizak et al., 2011a) and can glycosylate such heterologous proteins both in cell-based and cell-free systems (Kowarik et al., 2006a; Ollis et al., 2015).

An attractive feature of bacterial *N*-glycosylation systems is their inherent simplicity, which makes them readily amenable to reconstitution outside the cell. Indeed, following the functional expression of the *C. jejuni pgl* locus in *E. coli* cells, the same glycosylation reaction was recapitulated *in vitro* by Imperiali and coworkers who showed that purified *Cj*OST was capable of transferring a glycan from a synthetic donor, Und-PP-disaccharide, onto a synthetic peptide acceptor (Glover K. J. et al., 2005). Along similar lines, Aebi and coworkers described an *in vitro* glycosylation assay comprised of purified *Cj*OST, a purified acceptor protein, and LLOs bearing the *C. jejuni* heptasaccharide glycan (*Cj*LLOs) that were extracted from glycosylation-competent *E. coli* (Kowarik et al., 2006a). Using this greatly simplified and well-controlled *in vitro* system, they were able to evaluate the ability of *Cj*OST to glycosylate distinct folding states of a model acceptor protein, RNase A^S32D^, leading to important insights about the preferred conformation (folded vs. unfolded) of bacterial acceptor proteins and the timing (co- vs. post-translational) of the bacterial glycosylation process (Kowarik et al., 2006a). Further, despite being large, integral membrane proteins with 13 transmembrane segments (Lizak et al., 2011b), bacterial OSTs can be readily overexpressed and purified from a recombinant host like *E. coli*, and the robust protocols for large-scale purification of *Cj*OST and other bacterial OST homologs are documented (Jaffee and Imperiali, 2013; Jaroentomeechai et al., 2017). Taken together, these developments have established *in vitro* glycosylation as one of the standard tools in bacterial glycobiology and glycoengineering.

Building on these advances, Guarino and DeLisa explored coupling bacterial-based *in vitro* glycosylation with *E. coli*-based cell-free protein synthesis (CFPS) technology (Guarino and Delisa, 2012). Specifically, they demonstrated that by supplementing either standard cell-free S30 extracts derived from *E. coli* or the PURE (protein synthesis using recombinant elements) system (Shimizu et al., 2001) with purified *Cj*OST and extracted *Cj*LLOs, it was possible to achieve efficient glycosylation of different model glycoprotein targets including the *C. jejuni* AcrA protein and a single chain fragment variable (scFv) antibody engineered with a C-terminal glycosylation sequon. More recently, the DeLisa and Jewett groups have developed a more integrated, single-pot platform for cell-free glycoprotein synthesis (CFGpS) (Figure 3B, Scheme ii) in which S30 extracts were selectively enriched with both *Cj*OST and *Cj*LLOs, effectively bypassing the need for purification and extraction, respectively, of these essential glycosylation components (Jaroentomeechai et al., 2018). When these glyco-enriched extracts were supplemented with plasmid DNA encoding different acceptor proteins including human erythropoietin, protein synthesis and *N-*glycosylation were co-activated in a manner that resulted in appreciable amounts of site-specifically modified target proteins that retained biological activity. Importantly, the system was demonstrated to be highly modular, allowing several different *Cj*OST homologs and structurally-distinct glycans including the eukaryotic trimannosyl core glycan, Man_3_GlcNAc_2_, to be rapidly interchanged into the cell-free reaction. DeLisa, Jewett, and coworkers have recently extended the CFGpS platform for cell-free conjugate vaccine synthesis (Stark et al., 2019), which takes advantage of the fact that *Cj*OST has a relaxed glycan substrate specificity and is capable of catalyzing transfer of O-PS antigens to yield conjugate vaccines (Feldman et al., 2005; Terra et al., 2012). By developing S30 extracts from low-endotoxin *E. coli* cells expressing *Cj*OST and different O-PS structures, it was possible to decorate a panel of different FDA-approved protein carriers such as CRM197 and *Haemophilus influenza* protein D with pathogen-specific polysaccharides including the *O*-PS antigen from the highly virulent pathogen *Franciscella tularensis* subsp. *tularensis* (type A) strain Schu S4. Importantly, conjugates supplied by this cell-free technology were observed to elicit O-PS-specific antibodies and provided complete protection against pathogen challenge in immunized mice (Stark et al., 2019).

It should be pointed out that while the CFGpS systems described above rely on heterologous expression of OSTs and LLOs in cells *prior* to extract preparation, it should be possible to streamline these systems with cell-free biosynthesis of each glycosylation component. To this end, it has been demonstrated that full-length and active membrane-bound bacterial OSTs could be directly synthesized in cell-free extracts that were supplemented with nanodisc scaffolds (Schoborg et al., 2018). It has also been shown that chemically-defined LLOs bearing the *C. jejuni* glycan can be generated by *in vitro* assembly of a biosynthetic pathway comprised of purified GTs (Glover K. et al., 2005). By integrating the biogenesis of OSTs and LLOs *in vitro* with cell-free glycoprotein synthesis platforms, we anticipate the creation of a simplified yet highly modular framework for furthering the study and exploitation of the bacterial glycosylation mechanism.

In addition to *N*-linked glycosylation, certain bacterial species including those in *Neisseria* and *Pseudomonas* genera possess *O*-linked protein glycosylation pathways that involve a similar *en bloc* glycan transfer mechanism (Faridmoayer et al., 2007). Utilizing cell-free reconstitution, a central enzyme in this pathway, *O*-OST, was found to have extremely broad substrate promiscuity, both in terms of the recognizable glycan structures and their lipid carriers (Faridmoayer et al., 2008; Musumeci et al., 2013). These features make this class of OST enzymes especially attractive for biotechnological and biomedical applications. However, widespread use of *O*-OSTs for preparing useful glycoproteins has been hindered by the lack of a consensus glycosylation motif, which in turn limits our ability to perform *O*-glycosylation on heterologous targets. This hurdle was partially resolved recently with the rational design of a minimum optimal *O*-linked recognition (MOOR) motif that was recognized by the *O*-OST PglL from *Neisseria meningitidis* (Pan et al., 2016). The MOOR sequence, which is composed of 8 amino acids flanked by two hydrophilic motifs, was used to produce an *O*-linked conjugate vaccine against *Shigella flexneri*. Since *O*-OSTs can transfer a wide range of structurally-diverse *O*-polysaccharides (Faridmoayer et al., 2008), the advent of the MOOR motif is expected to accelerate the use of *O*-OST-based glycosylation as a platform to produce and engineer conjugate vaccines against diverse bacterial pathogens. In an important first step toward cell-free *O*-glycoprotein biosynthesis, the DeLisa group has generated S30 extracts enriched with different *O*-OSTs and LLOs bearing short-chain human *O*-glycans (e.g., Tn antigen, T antigen, and sialylated versions of both) (Natarajan et al., 2020). The resulting glyco-enriched extracts were capable of synthesizing antigenically authentic glycoforms of human mucin 1 (MUC1), thereby providing a platform for construction of designer *O-*glycoproteins and further expanding the cell-free glycoprotein expression toolkit.

#### GT-Mediated Protein Glycosylation and Glycan Elaboration

Processive protein glycosylation is prevalent in nature with the archetype represented by vertebrate mucin-type *O*-glycosylation, a mechanism whereby the glycan is assembled directly on the protein by sequential addition of monosaccharides by GTs (Hang and Bertozzi, 2005). Mucin-type *O*-glycosylation is initiated by the formation of α-glycosidic bonds between GalNAc monosaccharides and Ser/Thr residues that are catalyzed by a specific enzyme in the polypeptide-*N*-acetylgalactosaminyl transferase (ppGalNAcT) family. This core structure, named Tn antigen, can then be extended *via* sequential addition of other monosaccharides including Gal, GlcNAc, and Neu5Ac by one or more of the ~30 different Golgi-resident GTs (Bennett et al., 2012). Since mucin-associated *O*-glycan structures are associated with many types of cancer (Pinho and Reis, 2015), there is great interest in obtaining structurally-defined *O*-glycoproteins for the development of carbohydrate-based cancer vaccine candidates. One promising avenue has been chemoenzymatic synthesis for preparing large glycopeptides carrying cancer-related *O-*glycans including Tn and sialylated Tn antigens. For example, Clausen and coworkers pre-synthesized MUC1 peptides that were subsequently modified by a series of ppGalNAcT enzymes with differential glycosylation site preferences (Sorensen et al., 2006). The GalNAc moieties on the MUC1 glycopeptides were then elongated to T or sTn antigen using β3-Gal or ST6GalNAcI transferases, respectively (Figure 3B, Scheme iii). The resulting *O*-glycosylated mucin peptides were subsequently used to immunize mice, leading to the elicitation of Tn/sTn antigen-specific antibodies that could recognize specific types of cancer cells. This study highlights the potential of cell-free glycoprotein synthesis approaches in the design and production of carbohydrate-based vaccine candidates. It is worth mentioning that while *O*-GalNAcylation of mucin has been the focus of intense research, many lesser studied types of *O*-linked glycosylation have been reported in recent years including the enzymatic transfer of GlcNAc, Man, Fuc, Glc, Gal, and Xyl sugars onto specific proteins such as Notch receptors and epidermal growth factor like (EGF) repeats (Bennett et al., 2012; Haltiwanger et al., 2015; Varki, 2017a; Holdener and Haltiwanger, 2019). Given our incomplete understanding of these relatively new types of *O*-glycosylation, it stands to reason that cell-free approaches will help to decipher their mechanisms and roles in biology.

Certain gram-negative γ-proteobacteria have been found to contain unique processive *N*- and *O*-linked protein glycosylation pathways (Ohuchi et al., 2000; Zhou and Wu, 2009). Among them, *N*-glycosyltransferase (NGT) from *Actinobacillus pleuropneumoniae* (*Ap*NGT) is the best characterized enzyme, which is capable of transferring Glc residues onto the same Asn-X-Ser/Thr motif used in canonical *N*-glycosylation that proceeds by the *en bloc* mechanism (Choi et al., 2010; Kawai et al., 2011; Naegeli et al., 2014). The *Ap*NGT has been functionally transferred into *E. coli* (Naegeli et al., 2014), providing a novel mode of bacterial glycoengineering (Keys et al., 2017). Indeed, several groups have leveraged *Ap*NGT to site-specifically *N*-glycosylate target proteins with a Glc moiety that serves as a “glycan primer” for further extension to defined glycoforms such to α-Gal, lactose, siallylactose, LacNAc, and Lewis-X structures by prescribed GTs (Kightlinger et al., 2019; Tytgat et al., 2019). In the work by Aebi, Keys and coworkers, multiple copies of these glycoepitopes could then be installed on the same target protein to create multivalent glycopolymers or equipped onto self-assembling polypeptides to produce megadalton glycoprotein assemblies (Tytgat et al., 2019). Such multivalent glycostructures could find applications in antibody discovery and the development of novel biomedical materials. The Jewett and DeLisa groups devised a multi-pot reaction scheme whereby each pot contained *E. coli* extract synthesizing a specific GT enzyme (Kightlinger et al., 2019). These reaction pots containing active GTs could then be combined in a sequence-specific manner to prototype designer glycosylation pathways (Figure 3B, Scheme iii). This modular technology, called glycosylation pathway assembly by rapid *in vitro* mixing and expression (GlycoPRIME), enabled the generation of 23 unique glycan epitopes whose pathways were successfully transferred into *E. coli* to bio-manufacture useful glycoproteins including the H1HA10 protein vaccine containing an α-Gal epitope. This study showcases the power of cell-free synthetic glycobiology as a versatile tool to design, build, test, and employ designer glycosylation pathways for the development and production of putative glycomedicines. It should be pointed out that while unique bacterial enzymes such as *Ap*NGT have been harnessed for processive glycan construction, similar strategies have been developed using *Cj*PglB. That is, even though *Cj*PglB is known for its ability to transfer preassembled glycan structures, it can also be used to install a single GlcNAc residue onto acceptor protein targets as was shown recently by Liu et al. (2014). These authors designed a series of short polyisoprenol variants that were modified with a single GlcNAc monosaccharide and used these unnatural sugar-unnatural lipid conjugates to demonstrate that purified *Cj*OST could catalyze the formation of GlcNAc-ylated peptides and proteins. They further showed that these GlcNAc-ylated species could be extended with ENGases and GTs (e.g., EndoA, β1,4-GalT) thereby demonstrating a novel *in vitro* route to tailor-made glycoproteins.

## High-Throughput Screening Strategies For Improving Glycoenzymes

In the past few decades, directed evolution approaches have proven tremendously useful in improving and/or altering the activities of existing enzymes (Porter et al., 2016; Arnold, 2018). In any directed evolution experiment, the development of a reliable high-throughput screening (HTS) assay is critical to successful library-based isolation of enzyme candidates with desirable traits (Qu et al., 2020). Generally, any enzymatic reaction amenable to the use of chromo-, radio-, or fluorogenic substrates can be easily integrated into a HTS format using a standard multi-well plate. A direct coupling between enzymatic activity and signal generation has often been used to screen for GH activity using a chemical reporter substrate (Kwan et al., 2015). However, such direct coupling methods have been proven to be extremely difficult to adapt for screening libraries of glycoenzymes like GTs and OSTs, since glycosidic bond formation does not provide any convenient readouts (Chao and Jongkees, 2019). To circumvent this issue, numerous indirect coupling assays measuring signal from interactions between glycomolecule products or reaction byproducts with a secondary reporter have been developed. For example, the UDP-Glo assay from Promega measures luminescent signals generated from the coupling that occurs between the UDP byproduct of the GT reaction with ATP generation (Zegzouti et al., 2016). Aside from indirect coupling with NDP byproducts, most assays rely on the affinity of biomolecules, such as lectins or antibodies, toward glycans and glycoprotein products. These affinity reagents are often conjugated with a chemical reporter or with an enzyme such as horseradish peroxidase that can generate a spectroscopic signal upon the addition of specific chemicals. The concept of affinity-based indirect coupling has been widely applied for the screening of GT and OST enzymes in various formats including by enzyme-linked immunosorbent assay (Ihssen et al., 2012; Pandhal et al., 2013), glycophage display (Celik et al., 2010; Durr et al., 2010), fluorescence-activated cell sorting (FACS) assay (Aharoni et al., 2006; Glasscock et al., 2018), and modified colony blotting methods (Ollis et al., 2014).

Alternatively, mass spectrometry-based high-throughput screening (MS-HTS) is an emerging technology offering a rapid, label-free, quantitative, and highly sensitive method to screen biomolecule libraries (Xu et al., 2007). In addition to its well-established workflow, its ability to multiplex and to be integrated with other *in vitro* techniques makes MS-HTS an attractive tool to screen glycoenzyme libraries. Recent glycoengineering work by Mrksich and coworkers reported a novel MS-HTS strategy for the characterization of GT enzymes produced directly from CFPS lysate (Kightlinger et al., 2018). This platform, called glycosylation sequence characterization and optimization by rapid expression and screening (GlycoSCORES), integrates *E. coli*-based CFPS with self-assembled monolayers for matrix-assisted desorption/ionization (SAMDI) mass spectrometry. Specifically, by combining rapid *in vitro* biosynthesis of GTs, such as *Ap*NGT, in cell-free extract with high-throughput analysis of their activity using SAMDI-MS, the authors were able to systematically investigate the enzyme's substrate specificity using 3,480 unique peptides and 13,903 unique reaction conditions, revealing the optimal glycosylation sequon (Kightlinger et al., 2018). More recently, the same team extended the methodology to the analysis of intact glycoproteins (Techner et al., 2020), providing an exciting new avenue for the discovery and improvement of glycosylation enzymes. In addition, they used conditionally orthogonal peptide acceptor specificities of NGTs to site-specifically control installation of multiple distinct glycans (Lin et al., 2020).

## Concluding Remarks

The impact of glycomolecules in basic biology and applied biotechnology is undeniable. As such, new and expanded toolkits are required to help transform the field of glycoscience and realize its full potential across biology, chemistry, and material science (Walt, 2012). Methods for the synthesis, characterization, evolution, and database processing of glycomolecules and glycoenzymes are still lacking, especially in comparison to those available to scientists and engineers for studying and engineering nucleic acids and proteins. In this review, we have outlined a number of emerging cell-free synthetic glycobiology approaches for the biosynthesis of chemically-defined glycomolecules. The past decade has seen considerable improvements in one-pot multienzyme synthesis (OPME) platforms, yielding several synthesis modules specific for sugar nucleotide building blocks including those with non-canonical and non-natural structures. Many of these modules have been employed to construct remarkably diverse glycans, glycolipids, and glycopolymers such as heparin sulfate. In parallel, glycochemists have made significant progress integrating enzymatic glycan biosynthesis with automated solid-phase synthesis with the goal of offering a fully-commercialized machine capable of synthesizing pre-designed, complex glycans for structural and functional investigation.

Glycoproteins with structurally uniform glycoforms are highly valuable as research reagents and biotherapeutics. Yet, our progress in understanding the structure-activity relationships of glycoproteins has been hindered, due in large part to technical barriers in preparing glycoproteins bearing well-defined glycan structures. *In vitro* chemoenzymatic approaches using ENGases have emerged as a versatile strategy for assembling homogenous glycoproteins including, and perhaps most notably, antibodies with specific *N*-glycoforms. Such antibodies have been instrumental in gaining insight into how specific glycan epitopes influence the effector functions of therapeutic antibodies. Alternatively, CFPS combined with the power of cell-free synthetic biology and bacterial glycoenzymes such as OSTs and NGTs provides a fully-integrated platform for rapidly producing uniform glycoproteins by seamlessly integrating transcription/translation with protein glycosylation in a one-pot reaction.

From a cell-free synthetic biology perspective, remaining challenges include improving current cell-free systems to produce correctly folded versions of more complex proteins and protein complexes, expanding the genetic code of CFPS for the incorporation of multiple non-natural amino acids in a protein for site-selective modification, and to further optimize synthesis efficiency, product titers, and cost reduction. In addition, cell-free extract equipped with the machineries for protein glycosylation and other important post-translational modifications such as phosphorylation and acetylation will be increasingly in demand, especially by those working to fully expand our understanding of glycosylation networks and their regulation (Yu and Chen, 2016). From a glycobiology standpoint, one of the key limitations is the availability of glycoenzymes with high catalytic activity and ease of purification. Currently, only a small fraction of useful glycoenzymes has been cataloged, characterized, and commercialized for broader use (Walt, 2012), while the list of available glycoenzymes continues to expand (Moremen et al., 2018). Another potential challenge is the strict substrate specificity of glycoenzymes, which in turn limits their utility in biotransformation. Novel directed evolution and high-throughput screening methods leveraging the power of cell-free biology are needed to identify more efficient glycoenzyme variants with precisely tailored substrate specificity. Finally, integration of the tools from cell-free synthetic glycobiology with those from other disciplines including metabolic engineering (Wratil and Horstkorte, 2017); (Agatemor et al., 2019), mathematic modeling (Umana and Bailey, 1997; Spahn et al., 2016; Wayman et al., 2019), and machine learning and bioinformatics (Li F. et al., 2015; York et al., 2019) will be needed to solve the most complex problems in glycoscience. Only when these unmet needs have been addressed can the full potential of cell-free synthetic glycobiology be unlocked for furthering glycoscience and its application.

## Author Contributions

TJ, MT, ML, AA, NA, SC, MJ, and MD contributed to the writing and editing of the manuscript. All authors read and approved the final manuscript.

## Conflict of Interest

MD has a financial interest in Glycobia, Inc., Versatope, Inc., and Swiftscale Biologics, Inc. MD's interests are reviewed and managed by Cornell University in accordance with their conflict of interest policies. MJ has a financial interest in StemLoop, Swiftscale Biologics, Inc., and Design Pharmaceuticals. MJ's interests are reviewed and managed by Northwestern University in accordance with their conflict of interest policies. The remaining authors declare that the research was conducted in the absence of any commercial or financial relationships that could be construed as a potential conflict of interest.
